# Is Antibiotic Prophylaxis Needed for the Extraction of Premolars for Orthodontic Purposes?

**DOI:** 10.7759/cureus.57387

**Published:** 2024-04-01

**Authors:** Aditya Hurkat, Vinod K Krishna, Murugesan Krishnan

**Affiliations:** 1 Oral and Maxillofacial Surgery, Saveetha Dental College and Hospitals, Saveetha Institute of Medical and Technical Sciences, Saveetha University, Chennai, IND

**Keywords:** prophylaxis, therapeutic interventions, infection, orthodontic extraction, antibiotics

## Abstract

Introduction

Antibiotic prophylaxis for tooth extractions is a common practice in dentistry to prevent postoperative infections. However, the routine use of antibiotics has been questioned due to concerns about bacterial resistance and potential side effects. This study aimed to evaluate the necessity of postoperative antibiotics in patients undergoing orthodontic tooth extraction.

Materials and methods

This prospective study involved 100 patients requiring orthodontic tooth extraction, divided into two groups. The patients were recruited from Saveetha Dental College and Hospital, Chennai, India, after obtaining approval from the Institutional Human Ethics Committee, Saveetha Dental College (approval number: IHEC/SDC/OMFS-2103/23/293). Group 1 (n = 50) received antibiotics (amoxicillin 500 mg, three times a day for three days) after extraction, while Group 2 (n = 50) did not receive antibiotics. Postoperative infection was assessed on postoperative days (POD) 3 and 7. Data analysis was conducted using IBM SPSS Statistics for Windows, version 26.0 (released 2019, IBM Corp., Armonk, NY). Categorical variables were presented as frequencies and percentages, and differences between groups were assessed using chi-square or Fisher's exact tests. A p-value of <0.05 was considered statistically significant.

Results

The incidence of postoperative infection was recorded in both groups. In group 1 at POD 3 and POD 7, there were two patients and one patient with infection, respectively. In group 2 at POD 3 and POD 7, there were four patients and two patients with infection, respectively.

Conclusion

The findings of this study suggest that the routine administration of antibiotics for the non-traumatic extraction of teeth in healthy patients might not be necessary. The absence of postoperative infections in patients who did not receive antibiotics indicates that antibiotics may be avoidable in many cases of orthodontic tooth extraction. These results emphasize the importance of reconsidering the widespread use of antibiotics to combat the growing concern of bacterial resistance. Antibiotics should be prescribed judiciously, only for patients with specific medical conditions who are prone to infection. One of the limitations of this study is the limited sample size; hence, studies with larger and heterogeneous groups should be done to validate the same.

## Introduction

Antibiotic prophylaxis is a commonly employed strategy in dentistry to prevent potential postoperative infections following tooth extractions [1-3]. The rationale behind this practice is to eliminate or inhibit the growth of bacteria responsible for infections, thereby reducing the risk of complications and promoting favorable outcomes. However, the widespread and indiscriminate use of antibiotics has raised concerns regarding antimicrobial resistance and the potential adverse effects associated with their use [4-6]. Such issues have prompted researchers and healthcare professionals to reevaluate the necessity of routine antibiotic prescription in various dental procedures, including tooth extractions.

While antibiotics have proven effective in treating bacterial infections, their unnecessary use contributes significantly to the global problem of antimicrobial resistance [7]. The emergence of resistant bacterial strains challenges the effectiveness of commonly used antibiotics, limiting treatment options for serious infections and potentially leading to higher morbidity and mortality rates [8]. Consequently, antimicrobial stewardship and judicious antibiotic prescription have become critical priorities in dental practice [9].

Tooth extraction, particularly in orthodontic patients, is a common dental procedure [6]. Considering the prevalence of this intervention, it is essential to determine whether postoperative antibiotic prophylaxis is indeed warranted in such cases. Several studies have addressed this question, with some advocating for antibiotic prescription in specific high-risk scenarios, while others suggest a more conservative approach [10-12]. However, evidence-based guidelines for the use of antibiotics in orthodontic tooth extraction remain limited, and further research is needed to clarify the optimal approach.

This study aims to contribute to the existing body of knowledge by prospectively evaluating the need for postoperative antibiotics in patients undergoing orthodontic tooth extraction. By comparing the incidence of postoperative infections in a group receiving antibiotics with a group that does not, we seek to provide valuable insights into the appropriateness and potential benefits of antibiotic prophylaxis in this dental setting.

## Materials and methods

Study design and participants

This is a prospective clinical study. The G power calculation was done, and based on a 95% confidence interval, 96 was the total sample size achieved considering dropouts from the study. Four more patients were added, and the total sample size attained was 100 participants. The patients were recruited from Saveetha Dental College and Hospital after obtaining approval from the Institutional Human Ethics Committee, Saveetha Dental College (approval number: IHEC/SDC/OMFS-2103/23/293). Patients requiring extraction for orthodontic correction, patients with no comorbidities, and patients with an age range of 15-30 years were included in the study. Patients with any systemic disorders and patients with impacted teeth were excluded from the study. All the patients underwent oral prophylaxis before the extraction.

Randomization and blinding

The participants were randomly assigned to two groups using computer-generated random numbers. Group 1 (n = 50) received postoperative antibiotics, while Group 2 (n = 50) did not receive antibiotics. To ensure blinding, the study medications were dispensed in identical packaging, and patients, care providers, and outcome assessors were unaware of the assigned groups.

Intervention

Group 1 patients were prescribed amoxicillin 500 mg three times a day for three days, starting immediately after the tooth extraction. Group 2 patients did not receive any postoperative antibiotics.

Orthodontic Tooth Extraction Procedure

**Figure 1:**
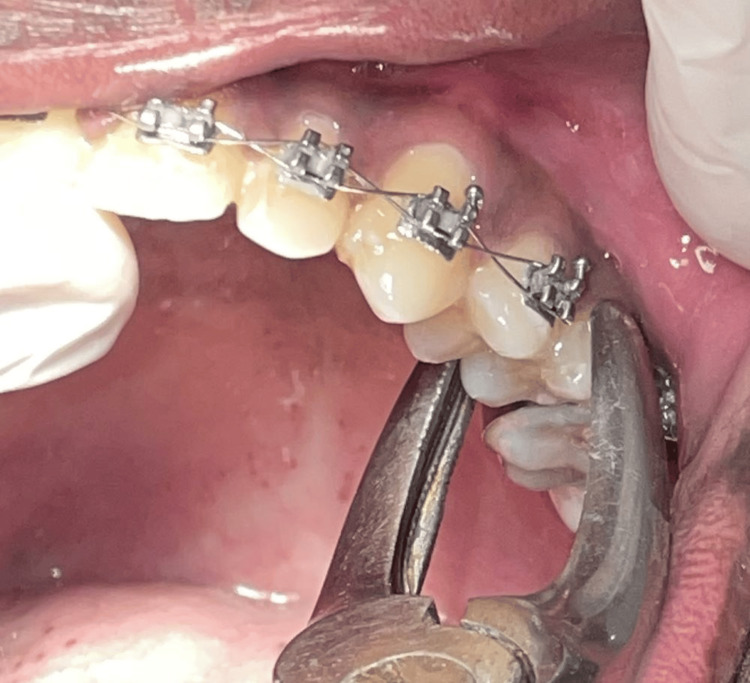
Upper premolar extraction

**Figure 2:**
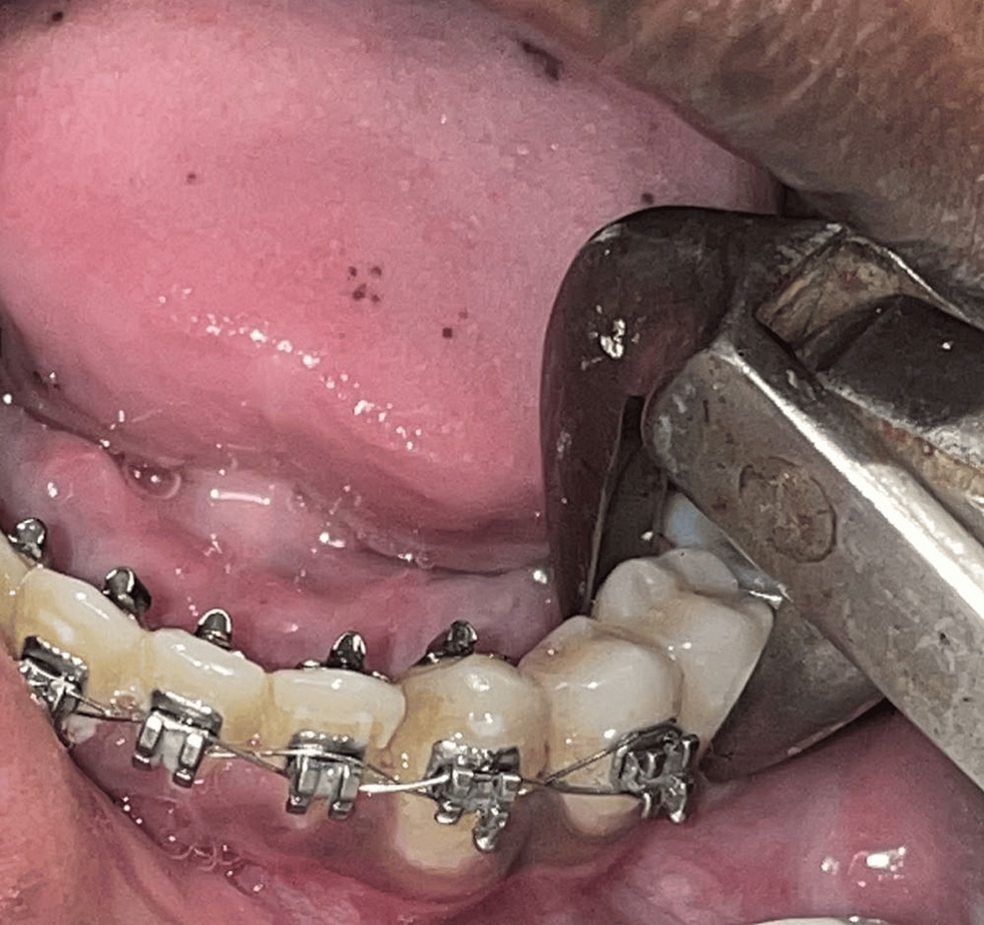
Lower premolar extraction

All tooth extractions (bicuspids of either side) were performed by experienced dentists or oral surgeons following standard aseptic techniques. Local anesthesia was administered using lidocaine with adrenaline (1:80,000) for pain control. Extraction was carried out using upper or lower premolar forceps atraumatically (Figures 1, 2). Following extraction, gauze was placed at the extraction site, and patients were instructed to maintain gentle pressure on the gauze for 30 minutes to minimize bleeding. For group 1 patients, antibiotics were prescribed, and for group 2 patients, no antibiotics were prescribed.

Outcome assessment

Postoperative infection was the primary outcome assessed in this study. Patients were evaluated for signs of infection on POD 3 and POD 7. Infection was defined as the presence of localized swelling, redness, purulent discharge, persistent pain at the extraction site, or any adverse reaction to the antibiotic. Patients who developed postoperative infections were appropriately managed with additional treatment.

Statistical analysis

Data analysis was conducted using appropriate statistical software. Categorical variables were presented as frequencies and percentages, and differences between groups were assessed using chi-square or Fisher's exact tests. A p-value of <0.05 was considered statistically significant.

## Results

A total of 100 patients were included in the study, with 50 patients randomized into Group 1 (received antibiotics) and 50 patients into Group 2 (did not receive antibiotics). 

The mean age in the control group was 25.1 ± 2.98 years, and in the case group, it was 23.4 ± 3.04 years. There were 50% males and 50% females in the control group and 33.3% males and 66.7% females in the case group (Table 1).

**Table 1:** Demographic data

Variable	Category	Control	Case	p-value
Median age (in years)	--	25.1 ± 2.98	23.4 ± 3.04	0.72
Gender	Male	25 (50%)	15 (33.3%)	0.63
	Female	25 (50%)	35 (66.7%)	0.70

Postoperative infection rates

**Figure 3:**
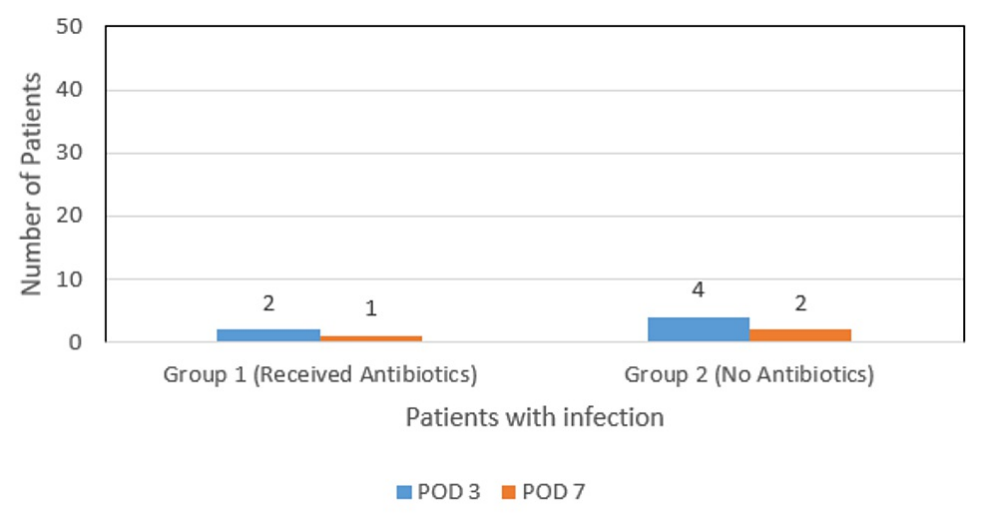
Incidence of infection POD: postoperative day

Postoperative infection rates were assessed on two different postoperative days (POD 3 and POD 7) for Group 1 and Group 2. The results are summarized in Table 2 and Figure 3:

**Table 2:** Postoperative infection rates Chi-square test; POD: postoperative day; NS: non-significant difference

Postoperative day	Group 1 (received antibiotics)	Group 2 (no antibiotics)	p-value
POD 3	2/50 (4%)	4/50 (8%)	0.678 (NS)
POD 7	1/50 (2%)	2/50 (4%)	1.000 (NS)

In Group 1, on POD 3, two patients out of 50 (4%) had infections. Finally, on POD 7, one patient out of 50 (2%) was observed with postoperative infections. In Group 2 (no antibiotics), on POD 3, four patients out of 50 (8%) showed infections. On POD 7, two patients out of 50 (4%) exhibited postoperative infections.

There was a non-significant difference in the incidence of postoperative infections between the two groups at POD 3 and POD 7.

## Discussion

The findings of this prospective study on the necessity of postoperative antibiotics in patients undergoing orthodontic tooth extraction provide valuable insights into the appropriate use of antibiotics in dental practice.

The study aimed to determine whether routine antibiotic prophylaxis is required following orthodontic tooth extraction, considering concerns over bacterial resistance and potential side effects associated with antibiotic use [1]. The results indicated that both Group 1 (received antibiotics) and Group 2 (did not receive antibiotics) demonstrated low postoperative infection rates, with no statistically significant difference between the groups. These findings are consistent with previous research that questioned the routine use of antibiotics in dental procedures [12,13].

The study's observation of minimal postoperative infection rates in Group 2 (no antibiotics) suggests that antibiotics may be avoidable in many cases of orthodontic tooth extraction. This supports the current trend in dental practice toward limiting the use of antibiotics to prevent increasing microbial resistance [14]. The avoidance of unnecessary antibiotic use is in line with antimicrobial stewardship principles aimed at preserving the effectiveness of antibiotics for future generations [5].

Adverse effects of antibiotics

Throughout the research period, Group 1 individuals did not report any notable negative consequences associated with their usage of antibiotics.

The study results suggest that the routine administration of antibiotics for orthodontic tooth extraction may not be necessary. Both groups exhibited similar low postoperative infection rates, with no statistically significant difference between them.

The low incidence of postoperative infections in Group 2 (no antibiotics) indicates that, in many cases of orthodontic tooth extraction, antibiotics may be avoidable without compromising patient outcomes. This finding aligns with the growing concern about bacterial resistance to antibiotics and emphasizes the importance of judicious antibiotic use to preserve their efficacy.

Based on the results of this study, routine antibiotic prophylaxis for non-impacted orthodontic tooth extraction in healthy and medically non-compromised patients may not be warranted. The low incidence of postoperative infections in both groups suggests that antibiotics may be unnecessary in many cases. Thus, careful consideration and evidence-based prescribing of antibiotics are essential to mitigate the risk of antimicrobial resistance and minimize adverse effects.

Several studies have previously investigated the effectiveness of antibiotic prophylaxis in dental procedures. While prophylactic antibiotics have shown benefits in specific high-risk scenarios, their routine use in uncomplicated dental extractions may not be justified [15,16,17]. The results of this study align with the consensus that the selective use of antibiotics based on individual patient risk factors and procedural complexity is more appropriate than universal prescribing.

Furthermore, the absence of significant adverse effects related to antibiotic use in Group 1 highlights the safety of the intervention when used appropriately. However, it is essential to remember that any adverse effect, no matter how rare, should be carefully weighed against the potential benefits of antibiotic prophylaxis [18-21].

Limitations

The limitations of this study include the relatively small sample size and the exclusion of patients with underlying medical conditions like diabetes mellitus, infective endocarditis, patients on immunosuppressants, patients on anticoagulant therapy, and recent history of fever or infection.

## Conclusions

The results of this study suggest that routine antibiotic prophylaxis may not be necessary for non-impacted orthodontic tooth extraction in healthy patients. Antibiotics should be prescribed judiciously, considering individual patient factors and the potential risks of antimicrobial resistance. Evidence-based prescribing practices in dental care contribute to the global effort to mitigate bacterial resistance and optimize patient outcomes.
